# ALKBH4 Stabilization Is Required for Arsenic-Induced 6mA DNA Methylation Inhibition, Keratinocyte Malignant Transformation, and Tumorigenicity

**DOI:** 10.3390/w14223595

**Published:** 2022-11-08

**Authors:** Yan-Hong Cui, Emma Wilkinson, Jack Peterson, Yu-Ying He

**Affiliations:** 1 Section of Dermatology, Department of Medicine, University of Chicago, Chicago, IL 60637, USA; 2 The College, Biological Science Division, University of Chicago, Chicago, IL 60637, USA

**Keywords:** arsenic, ALKBH4, 6mA DNA methylation, autophagy, skin cancer, epigenetics

## Abstract

Inorganic arsenic is one of the well-known human skin carcinogens. However, the molecular mechanism by which arsenic promotes carcinogenesis remains unclear. Previous studies have established that epigenetic changes, including changes in DNA methylation, are among the critical mechanisms that drive carcinogenesis. *N*^*6*^-methyladenine (6mA) methylation on DNA is a widespread epigenetic modification that was initially found on bacterial and phage DNA. Only recently has 6mA been identified in mammalian genomes. However, the function of 6mA in gene expression and cancer development is not well understood. Here, we show that chronic low doses of arsenic induce malignant transformation and tumorigenesis in keratinocytes and lead to the upregulation of ALKBH4 and downregulation of 6mA on DNA. We found that reduced 6mA levels in response to low levels of arsenic were mediated by the upregulation of the 6mA DNA demethylase ALKBH4. Moreover, we found that arsenic increased ALKBH4 protein levels and that ALKBH4 deletion impaired arsenic-induced tumorigenicity *in vitro* and in mice. Mechanistically, we found that arsenic promoted ALKBH4 protein stability through reduced autophagy. Together, our findings reveal that the DNA 6mA demethylaseALKBH4 promotes arsenic tumorigenicity and establishes ALKBH4 as a promising target for arsenic-induced tumorigenesis.

## 1. Introduction

Arsenic is a natural metalloid found in the earth’s crust and is widely found throughout the environment. In the environment, arsenic can bind with several elements, including oxygen, sulfur, and carbon; these molecules exist in air, water, and soil, as well as in the bacteria that live within soil and sediment [1–4]. Arsenic is water-soluble and can leach from the ground and contaminate neighboring bodies of water. Arsenic contamination has also been detected in other water sources, including rain and snow, and in discarded industrial wastes.

Although humans are exposed to multiple forms of arsenic, inorganic arsenic exposure poses the greatest risk to human health. Most human intake of arsenic occurs from the consumption of inorganic arsenicals found in drinking water ingested from contaminated bodies of water; such intake, therefore, poses a great public health issue globally. Exposure to acute, high levels of arsenic can lead to acute toxicity and even death. In contrast, exposure to chronic low doses of arsenic leads to a much different pathophysiology and can result in diseases such as cancer of the lungs, bladder, and skin [5–11]. Notably, a major target organ for arsenic exposure is the skin; exposure to chronic low levels of arsenic has been found to promote skin cancer, the most prevalent form of cancer.

The process by which arsenic is metabolized is crucial for understanding the pathophysiology of arsenic-induced toxicities. Arsenic metabolism is a well-studied process that is characterized by sequential reduction/oxidation and methylation reactions [12]. Arsenic is taken up and absorbed by cells as arsenate, the pentavalent form of arsenic (iAs^V^), and is reduced into the more toxic, trivalent form of arsenic, arsenite (iAs^III^), and arsenite is more rapidly absorbed by cells and slowly excreted, compared with arsenate and organic arsenic [12–15]. Arsenite is then oxidized and methylated, with methyl groups provided by S-adenosylmethionine (SAM), the universal methyl donor, into monomethylarsonic acid (MMA^V^) [12,13]. MMA^V^ is then reduced into monomethylarsonous acid (MMA^III^) and MMA^III^ is then subsequently oxidized/methylated into dimethylarsinic acid (DMA^V^), which can then be further reduced/methylated into dimethylarsinous acid (DMA^III^) [12,13]. Of these mono- and dimethyl intermediates, MMA^III^ and DMA^III^ are believed to be more toxic than their pentavalent forms [12].

Several mechanisms have been suggested to play crucial roles in arsenic tumorigenicity, including oxidative stress, DNA damage, and epigenetic dysregulation, stemming from cellular arsenic uptake and the generation of arsenic metabolites. Arsenic induces epigenetic dysregulation, including changes in DNA methylation, histone modifications, and epitranscriptomic changes, including changes in RNA methylation [16–19]. Epigenetic modifications are reversible modifications that play critical roles in gene expression. Arsenic can decrease the cellular availability of methyl groups and contribute to global DNA hypomethylation due to the depletion of SAM required for arsenic metabolism/methylation, therefore leading to the reduced methylation of DNA, RNA, and proteins [19–24]. Arsenic can also target the zinc-finger domains of the ten–eleven translocation (TET) proteins; these proteins play critical roles in regulating DNA methylation and histone modifications and can disturb the TET-mediated oxidation of DNA methylation in 5-methylcytosine (5mC) [25–27]. However, the mechanism by which arsenic contributes to epigenetic and epitranscriptomic dysregulation in skin cancer has remained unknown.

*N*^*6*^-methyladenine (6mA) is the most prevalent DNA methylation in prokaryotes and plays critical roles in the regulation of the restriction–modification (R–M) system, DNA replication and mismatch repair, transposition, transcription, and cellular protection [28–31]. In contrast, in eukaryotes, 5-methylcytosine (5mC) is the most abundant modified nucleotide in DNA [32–36]. Specifically, 5mC is a methylated form of the DNA base cytosine (C) that modulates gene transcription and has several critical roles in cancer biology [32,37,38]. However, in recent studies, the presence of DNA 6mA has been discovered in several eukaryotes, including *Chlamydomonas reinhardtii* [39], *ciliates* [40,41], *Caenorhabditis elegans* (*C. elegan*) [42], *Drosophila* [43], and fungi [44]. These previous studies have suggested that 6mA can potentially act as an alternative DNA methylation modification [45–48]. In addition, 6mA was shown to play crucial roles in stress response, including mitochondrial stress [49] and environmental stress in the mouse brain [48]. However, 6mA’s role in gene regulation and disease pathogenesis remains largely unknown. In particular, the role of 6mA modification in arsenic-induced skin tumorigenesis has not previously been explored.

A few 6mA writers and erasers have been identified in different organisms. In *C. elegans*, NMAD-1, which belongs to the MT-A70 family of demethylases, can serve as a DNA 6mA demethylase, or “eraser”, while DAMT-1 may serve as a DNA 6mA methyltransferase or “writer” [42,50]. In comparison, in mammalian systems, N6AMT1 and METTL4 are shown to be DNA 6mA writers [51,52]. In addition, in mammalian cells and invertebrates, ALKBH1 and ALKBH4 dioxygenases, which belong to the AlkB family of proteins, have also been identified as DNA 6mA erasers [47,51–53]. However, the regulation of 6mA remains largely enigmatic.

In this study, we investigated the role of 6mA and its eraser, ALKBH4, in arsenic-induced malignant transformation and tumorigenicity. We show that chronic low-level arsenic exposure upregulates ALKBH4 and downregulates DNA 6mA modification in keratinocytes. In addition, we found that ALKBH4 inhibition decreased arsenic-induced tumorigenesis. Moreover, we identified that arsenic promoted the stabilization of ALKBH4 via the inhibition of autophagy. Taken together, these results demonstrate that DNA 6mA modification and ALKBH4 act as a novel epigenetic mechanism within the arsenic damage response and tumorigenesis.

## 2. Materials and Methods

### 2.1. Cell Culture

HaCaT (human keratinocyte, kindly provided by Dr. Fusenig), MEF (mouse embryonic fibroblasts), and HEK-293T (human embryonic kidney) cells were maintained in Dulbecco’s modified Eagle’s medium (Invitrogen, Carlsbad, CA, USA) supplemented with 10% fetal bovine serum (Gibco), 100 U/mL penicillin, and 100 μg/mL streptomycin (Invitrogen, Carlsbad, CA, USA) as described previously [54,55].

### 2.2. Plasmid and Lentivirus Generation and Infection

Lentivirus was produced through co-transfection into HEK-293T cells with lentiviral constructs together with the pCMVdelta8.2 packaging plasmid and pVSV-G envelope plasmid using X-tremeGENE 9 as described previously. Virus-containing supernatants were collected at 24–48 h. Target cells were infected in the presence of polybrene (8 μg/mL) (Sigma-Aldrich, St. Louis, MO, USA) and selected with puromycin (Santa Cruz Biotechnology, Santa Cruz, CA, USA) at 1 μg/mL for 7 days [54,55].

### 2.3. Quantitative Real-Time PCR (qPCR)

Quantitative real-time PCR assays were performed using a CFX Connect real-time system (Bio-Rad, Hercules, CA, USA) with Bio-Rad iQ SYBR Green Supermix (Bio-Rad, Hercules, CA, USA) as described previously [54,55]. The threshold cycle number (CQ) for each sample was determined in triplicate. The CQ values for *ALKBH4* were normalized against *GAPDH* [54–56].

### 2.4. The 6mA Dot Blot Assay

The total DNA was isolated with a QIAamp DNA Mini Kit (QIAGEN, Hilden, Germany, cat: 51306) according to the manufacturer’s instructions. DNA was treated with RNase A for 1 h at room temperature followed by cleanup. DNA was then denatured by heating at 98 °C for 10 min, spotted on Amersham Hybond-N + membrane (GE Healthcare, Chicago, IL, USA), and subsequently UV-cross-linked twice to the membrane. After drying, the membrane was blocked with 5% BSA (in 1× PBST) for 1 h and then incubated with a specific anti-m^6^A antibody (Synaptic Systems, 202 003, 1:2000; Goettingen, Germany) overnight at 4 °C. Next, the membrane was incubated with HRP-conjugated anti-rabbit IgG (Cell Signaling Technology, Beverly, MA, USA) for 1 h at room temperature and then developed with a Thermo ECL SuperSignal Western Blotting Detection Reagent (Thermo Fisher Scientific, Waltham, MA, USA) as described previously [51,56].

### 2.5. Quantification of m^6^A, m^1^A, and Am Levels in mRNA by Ultra-High-Performance Liquid Chromatography–Tandem Mass Spectrometry (UHPLC-MS/MS) Assay

Quantitative analysis of mRNA modification levels was conducted as previously described [57,58]. Briefly, mRNA was extracted using a Dynabeads^™^ mRNA DIRECT^™^ Purification Kit (Thermo Fisher Scientific, Waltham, MA, USA), according to the manufacturer’s instructions. Samples were purified twice to remove rRNA contamination. mRNA was further purified and treated with DNAse using a ZYMO RNA Clean and Concentrator Kit (ZYMO, Irvine, CA, USA). Briefly, 50 ng of mRNA in 18 uL nuclease-free water was digested with nuclease P1 (Sigma, N8630) in 20 uL of a buffer containing 200 mM NH40Ac and incubated for 2 h at 42 °C. Then, 2.5 uL of 10× FastAP buffer and 1 uL FastAP enzyme (Thermo Fisher Scientific, Waltham, MA, USA) were added to the mixture and incubated at 37 °C for 2–4 h. The samples were then diluted 1:1 with nuclease-free water, filtered (0.22 mm, Millipore), and injected into a C18 reverse phase column coupled online to an Agilent 6460 QQQ LC-MS/MS spectrometer in the positive electrospray ionization mode. The nucleosides were quantified by using retention time and the nucleoside-to-base ion mass transitions. Quantification was performed by comparing against a standard curve obtained from pure nucleoside standards ran with the same batch of samples.

### 2.6. Immunoblotting

Protein extracts were obtained by washing cells once with cold PBS and then lysing cells in a RIPA buffer (Thermo Fisher Scientific, Waltham, MA, USA) supplemented with a protease and phosphatase inhibitor mixture (Thermo Fisher Scientific, Waltham, MA, USA). Protein samples were then sonicated and spun down at 13,200 RPM for 15 min at 4 °C. After quantifying protein concentrations using the Pierce BCA assay (Thermo Fisher Scientific, Waltham, MA, USA), the samples were heated for 10 min at 70 °C. Protein abundance was analyzed through SDS–polyacrylamide gel electrophoresis followed by immunoblotting. The antibodies used were as follows: anti-m^6^A (Synaptic system, 202 003, 1:2000); anti-ALKBH4 (Proteintech, 19822–1-AP, 1:1000); anti-GAPDH (Santa Cruz, sc-47724, 1:5000); and anti-β-actin (Santa Cruz, SC-47778, 1:5000).

### 2.7. Soft Agar Colony Formation and Cell Proliferation Assay

The soft agar assay was performed as described previously [56]. Briefly, cells (500 or 1000 cells) were suspended in 0.35% agar in 1XDMEM/10%FBS growth medium and seeded in 35 mm dishes pre-coated with 0.5% agar in 1X DMEM/10%FBS growth medium, followed by incubation at 37 °C with 5% CO_2_. Cells were fed 1–2 times per week with a cell culture medium. After 10–14 days, colonies were stained with 0.005% Crystal Violet for more than 1 h. For the cell proliferation assay, the number of cells was assessed with a Cell Counting Kit-8 (CCK-8) (Sigma-Aldrich, St. Louis, MO, USA) following the manufacturer’s protocol [55].

### 2.8. Tumorigenicity Assay in Immunocompromised Mice

All animal procedures were approved by the University of Chicago institutional animal care and use committee. Athymic nude mice were obtained from Harlan-Envigo. As-Tr cells (2 million) in PBS with or without ALKBH4 deletion were injected subcutaneously into the right flanks of female mice (6–8 weeks of age). Tumor growth was monitored and measured with a caliper, and tumor volume was calculated using the following formula: tumor volume (mm^3^) = d^2^ × D/2, where d and D are the shortest and the longest diameters, respectively [56].

### 2.9. Statistical Analyses

Statistical analyses were carried out using Prism 7 and 9 (GraphPad). Data are expressed as the mean of at least three independent experiments. Error bars indicate the SDs or SEs of the means. *p* < 0.05 was considered statistically significant.

## 3. Results

### 3.1. ALKBH4 Upregulated in Arsenic-Induced Skin Cancer

**Figure 1.**
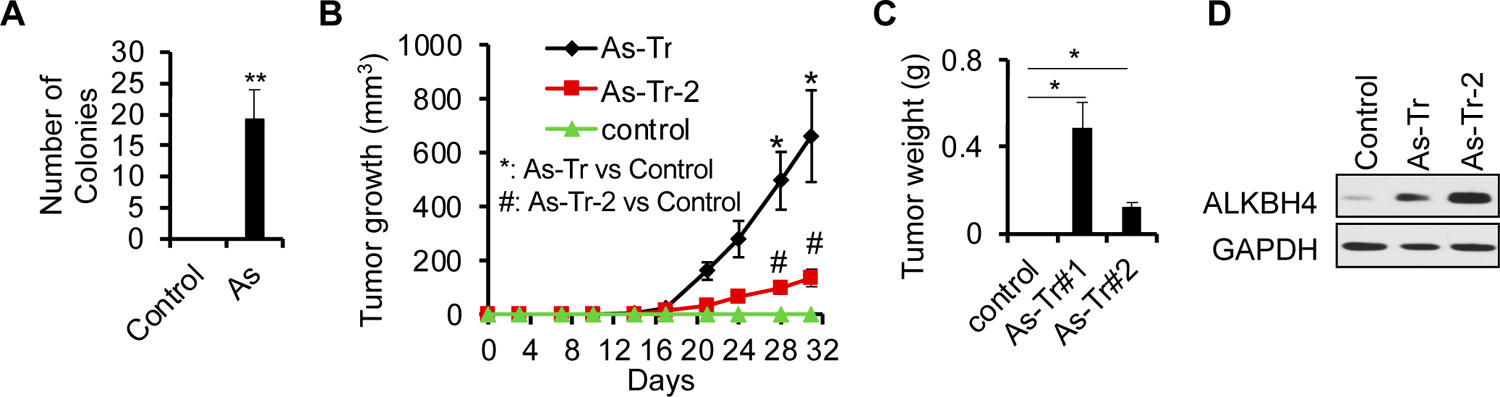
ALKBH4 upregulated in arsenic-induced transformation of keratinocytes: (**A**) soft agar assay of HaCaT cells with or without chronic low level of inorganic arsenite (As, 100 nM) treatment;(**B**,**C**) tumor volume (**B**) and weight (**C**) of control, As-Tr, and As-Tr-2 cells in nude mice (*n* = 3). Data are shown as mean ± SE (*n* = 3); (**D**) immunoblot analysis of ALKBH4 in control, As-Tr cells. GAPDH was used as a loading control. * and ^#^, *p* < 0.05, **, *p* < 0.01, compared with the control group; Student’s *t*-test.

To determine the epigenetic mechanism in arsenic-induced malignant transformation and tumorigenicity, we used a malignant transformation model induced by chronic arsenic exposure [7,55]. We continuously exposed HaCaT cells, a human keratinocyte cell line, to a physiologically relevant low level of inorganic arsenite (106277; EMD Millipore, 100 nM) for 28 weeks, thereby generating HaCaT cells with chronic arsenic damage [7,55]. Next, to evaluate whether treatment with 100 nM of inorganic arsenite for 28 weeks yielded malignant transformation, we performed soft agar colony assays. As expected, exposure to chronic low levels of arsenite in HaCaT cells induced colony formation only in the arsenic-treated cells but not in untreated cells (Figure 1A). Next, we isolated the cells from the soft agar colonies, referred to as arsenic-transformed cells (As-Tr). When inoculated into nude mice, As-Tr cells grew tumors, while control cells did not (Figure 1B,C). These findings are consistent with previous reports [7,59] using a different method of isolating cells and demonstrate that exposing HaCaT cells to a chronic low level of arsenic results in malignant transformation and tumorigenicity.

Next, we sought to identify the potential epigenetic differences across As-Tr and control cells. We found that the DNA 6mA demethylase ALKBH4 was upregulated in As-Tr cells, compared with control cells (Figure 1D). These findings demonstrate that chronic arsenic exposure induces malignant transformation and tumorigenesis in parallel with the upregulation of ALKBH4.

### 3.2. ALKBH4 Is Required for Arsenic-Induced Tumorigenicity

**Figure 2.**
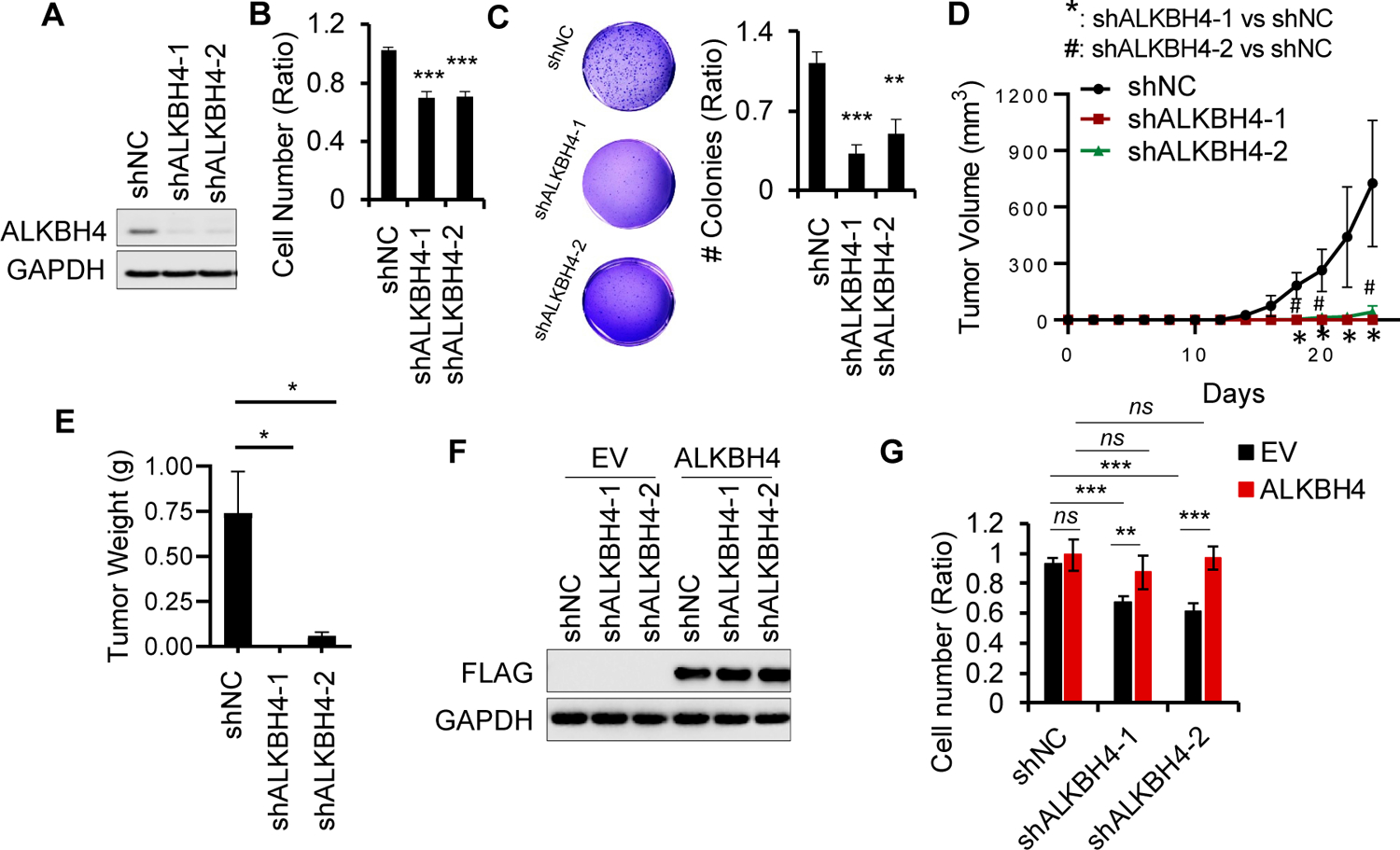
ALKBH4 is required for arsenic-induced malignant transformation and tumorigenicity: (**A**) immunoblot analysis to confirm ALKBH4 knockdown in As-Tr cells; (**B**) cell proliferation assay in As-Tr cells with or without ALKBH4 depletion; (**C**) soft agar assay in As-Tr cells with or without ALKBH4 depletion; (**D**,**E**) tumor volume (**D**) and weight (**E**) of As-Tr cells with or without ALKBH4 depletion in nude mice (*n* = 3); (**F**) immunoblot analysis to confirm ALKBH4 (FLAG) overexpression in As-Tr cells with or without ALKBH4 knockdown transfected with empty vector (EV) or construct overexpressing ALKBH4-FLAG; (**G**) cell proliferation assay in cells as in F. Data are shown as mean ± SD (*n* = 3). *, *p* < 0.05; **, *p* < 0.01; ***, *p* < 0.001; ns, not significant; compared with shNC group (**B**–**D**) or between comparison groups (**E**,**G**); Student’s *t*-test.

To determine the functional significance of ALKBH4 upregulation in arsenic-induced tumorigenicity *in vitro* and *in vivo*, we first performed cell proliferation and soft agar assays in As-Tr cells with or without ALKBH4 knockdown. We found that cell proliferation was significantly decreased after ALKBH4 inhibition (Figure 2A,B). In addition, ALKBH4 inhibition in As-Tr cells impaired anchorage-independent soft agar colony formation (Figure 2C). Furthermore, *in vivo*, we found that As-Tr cells with ALKBH4 knockdown displayed drastically reduced tumor growth (Figure 2D) and weight (Figure 2E) when injected into nude mice. Repletion with ALKBH4 rescued the effect of ALKBH4 knockdown on cell proliferation in As-Tr cells (Figure 2F,G). Notably, the overexpression of ALKBH4 in control As-Tr cells did not affect cell proliferation, suggesting that the high level of endogenous ALKBH4 expression saturated the cell proliferation capacity in As-Tr cells. Overall, these results clearly demonstrate that ALKBH4 is required for arsenic-induced malignant transformation and tumorigenicity and suggest that ALKBH4 is a tumor-promoting enzyme involved in arsenic-induced tumorigenesis.

### 3.3. ALKBH4 Upregulation Inhibits DNA 6mA Methylation in Arsenic-Transformed Cells

**Figure 3.**
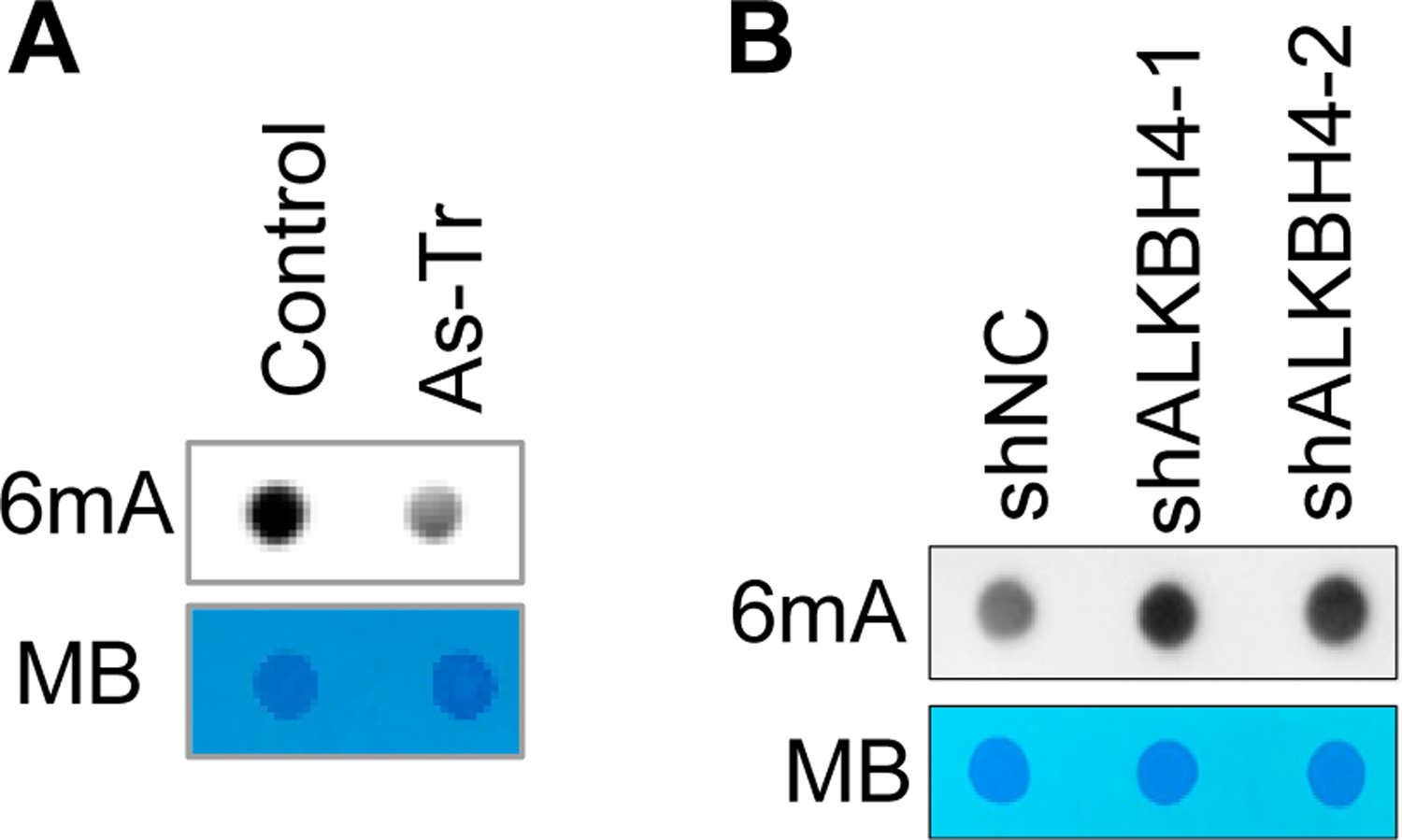
ALKBH4 upregulation in arsenic-exposed cells suppresses DNA 6mA methylation: (**A**,**B**) dot blot assays of DNA 6mA levels in control and As-Tr cells (**A**) and in As-Tr cells with or without ALKBH4 knockdown (**B**). Methylene blue (MB) staining was used as a loading control.

As ALKBH4 has been shown to demethylate DNA 6mA [52], we expected that arsenic-induced ALKBH4 upregulation would inhibit 6mA enrichment. Indeed, we found that DNA 6mA levels were downregulated in As-Tr cells, compared with control cells (Figure 3A). Furthermore, ALKBH4 knockdown increased DNA 6mA levels in arsenic-treated cells (Figure 3B). These results demonstrate that chronic arsenic exposure downregulates DNA 6mA methylation by upregulating ALKBH4.

### 3.4. ALKBH4 Does Not Regulate mRNA Modification of Arsenic Treatment Response

**Figure 4.**
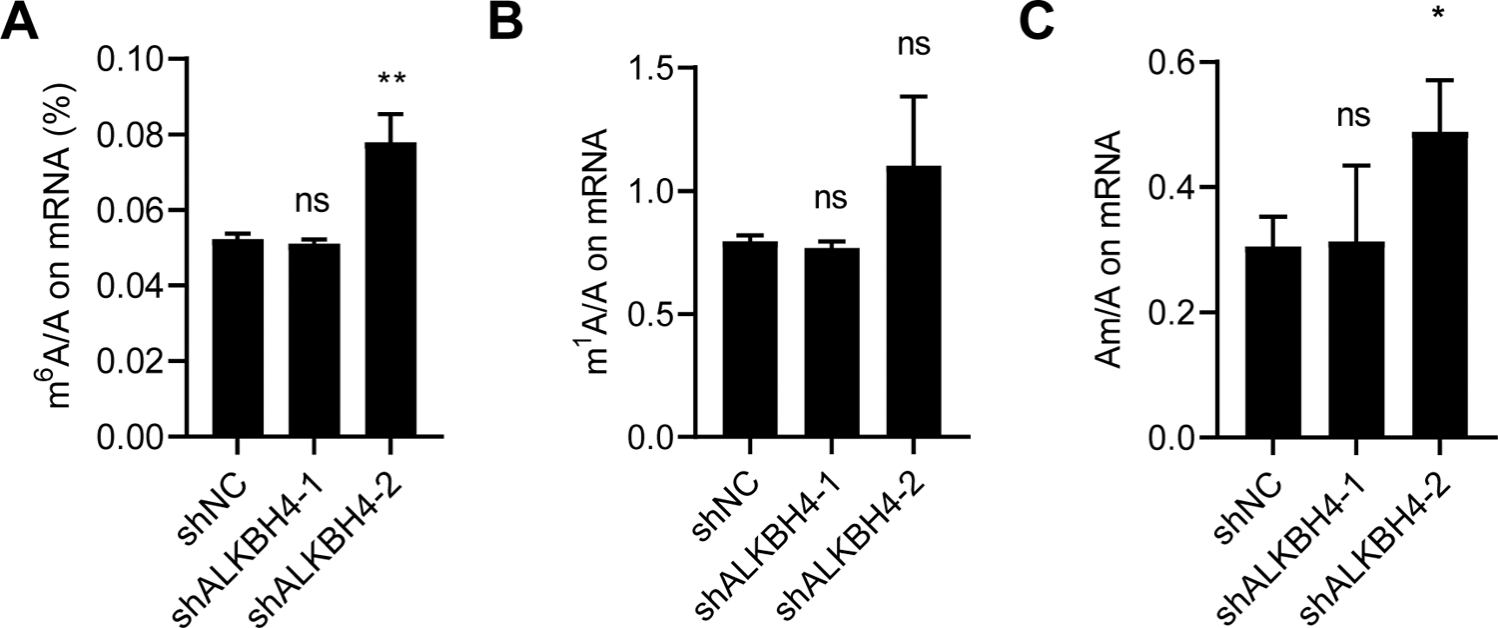
ALKBH4 does not affect m^6^A, m^1^A, or Am mRNA modifications on arsenic-transformed keratinocytes: (**A**–**C**) quantification of m^6^A/A (%) (**A**), m^1^A/A (a.u., arbitrary unit) (**B**) and Am/A (a.u., arbitrary unit) (**C**) ratios in polyadenylated RNA by UHPLC-MS/MS with or without ALKBH4 depletion in As-Tr cells. *, *p* < 0.05; **, *p* < 0.01; ns, not significant; compared with shNC group; Student’s *t*-test.

The mammalian AlkB family proteins consist of nine homologous enzymes (ALKBH1-8, FTO), which are derived from the prokaryotic DNA repair enzyme AlkB. The AlkB family depends on Fe^2+^ and α-ketoglutarate to catalyze the demethylation of different substrates, including double-stranded DNA, single-stranded DNA, mRNA, non-coding RNA, and proteins [60–63]. Our lab previously found that chronic arsenic exposure reduced *N*^*6*^-methyladenosine (m^6^A) mRNA methylation levels [55]. Here, we sought to determine whether ALKBH4 also regulates mRNA m^6^A methylation levels in As-Tr cells. To this end, we comprehensively evaluated several mRNA modifications with or without ALKBH4 inhibition in As-Tr cells using liquid chromatography–mass spectrometry. We found that ALKBH4 knockdown did not consistently affect the levels of m^6^A, *N*^*1*^-methyladenosine (m^1^A), or 2-O-methyladenosine (Am) (Figure 4A–C). Taken together, these findings demonstrate that while ALKBH4 regulates DNA 6mA modification, it does not affect m^6^A, m^1^A, or Am mRNA modifications in arsenic-transformed cells.

### 3.5. ALKBH4 Protein Stability Is Upregulated by Arsenic via Autophagy Inhibition

**Figure 5.**
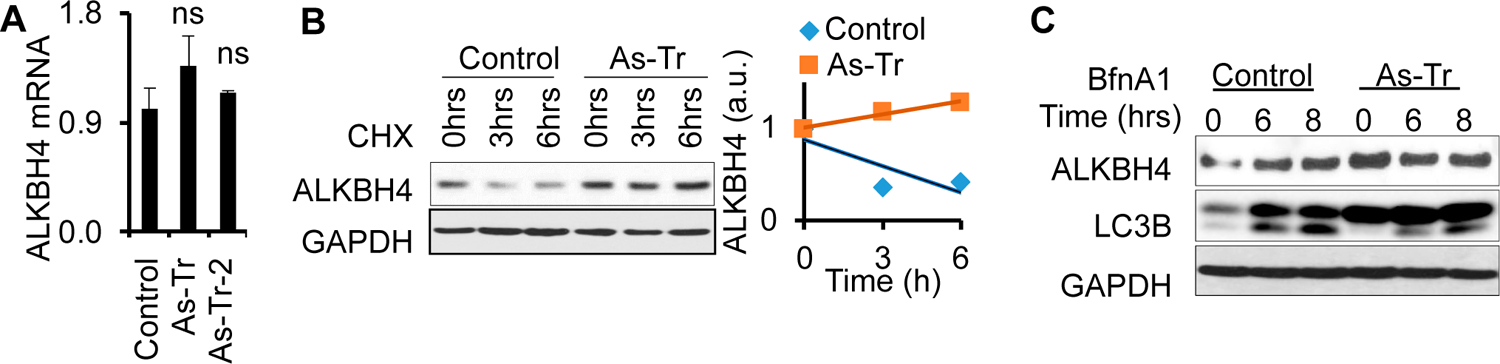
ALKBH4 stability is upregulated by arsenic via autophagy inhibition: (**A**) qPCR analysis of *ALKBH4* mRNA levels in control and As-Tr cells; (**B**) immunoblot analysis of ALKBH4 in control and As-Tr cells treated with cycloheximide (CHX, 100 μg/mL) over a time course; (**C**) immunoblot analysis of ALKBH4 following treatment with BfnA1 (50 nM) for 6 and 8 h in control and As-Tr cells. ns, not significant (**A**); compared with the control group; Student’s *t*-test.

To determine the mechanism by which arsenic upregulates ALKBH4 expression, we first assessed whether arsenic increases *Alkbh4* mRNA levels. We found that *Alkbh4* mRNA levels remained unchanged in As-Tr cells relative to control cells (Figure 5A). These results indicate that arsenic upregulates ALKBH4 protein but not *Alkbh4* mRNA levels.

To investigate how arsenic upregulates ALKBH4 protein, we assessed the effect of arsenic on ALKBH4 protein stability using a cycloheximide (CHX) protein chase assay over a six-hour time course. Compared with control cells, ALKBH4 protein stability was increased in As-Tr cells (Figure 5B), indicating that arsenic increases ALKBH4 protein stability. Next, we sought to determine the mechanism by which arsenic promotes ALKBH4 protein stability. Protein stability can be regulated by two major mechanisms: proteasomal degradation and autophagy. Our previous studies have shown that chronic low levels of arsenic exposure inhibit the autophagy pathway [55]. We suspected that ALKBH4 protein stability would also be regulated by autophagy dysfunction in arsenic-transformed cells. Using a lysosome inhibitor, bafilomycin A1 (BfnA1), we found that in the control cells, ALKBH4 protein levels were increased, while ALKBH4 protein levels remained unchanged in As-Tr cells (Figure 5C). Notably, control cells showed more LC3-II accumulation than As-Tr cells, suggesting that autophagy was inhibited in As-Tr cells, consistent with our previous work [55]. These findings suggest that arsenic increases ALKBH4 protein stability through the inhibition of the autophagy–lysosomal pathway.

## 4. Discussion

Chronic low-level arsenic exposure can induce malignant transformation and contribute to skin carcinogenesis. However, the mechanism by which arsenic induces skin cancer remains poorly understood. In this study, we showed that chronic low doses of arsenic exposure decrease 6mA DNA modification levels and increase ALKBH4 protein expression. We found that ALKBH4 as a DNA 6mA demethylase decreased DNA 6mA levels in arsenic-transformed keratinocytes. Moreover, we found that arsenic-induced tumorigenicity was decreased by ALKBH4 inhibition both *in vitro* and *in vivo*. Taken together, these results illustrate that ALKBH4, acting as a DNA 6mA demethylase, is critical for arsenic-induced skin tumorigenesis.

Our findings demonstrate that DNA 6mA and ALKBH4 act as a new epigenetic mechanism in arsenic-induced malignant transformation and tumorigenicity. Arsenic is a non-mutagenic human carcinogen that cannot directly induce DNA mutations. However, several pieces of evidence suggest that arsenic carcinogenicity results from epigenetic dysregulation, particularly in DNA methylation. Arsenic can activate oncogenes or silence tumor suppressor genes by regulating the methylation of genes [18,64–67]. In mammals, DNA methylation occurs on CpG and non-CpG sequences, CHG, and CHH (where H = C, T, or A). DNA methylation is catalyzed by a family of DNA methyltransferases (DNMTs) that transfer a methyl group from *S*-adenyl methionine (SAM) to the fifth carbon of a cytosine residue to form 5-methylcytosine (5mC) [18,33]. Previous studies have found that arsenic can inhibit DNMTs, which results in whole-genome and localized gene-specific demethylation [68,69]. To our knowledge, the present study is the first to evaluate the role of arsenic stress in regulating DNA 6mA modification. We found that DNA 6mA levels were decreased in arsenic-treated cells, compared with control cells. It is possible that arsenic may decrease DNA 6mA levels by depleting the cellular availability of methyl groups since the metabolism and methylation of inorganic arsenic requires S-adenosylmethionine (SAM) as a methyl donor [19–24]. However, further studies are needed to further elucidate the role of DNA 6mA in arsenic-induced skin cancer.

ALKBH4 is orthologous to the 6mA demethylases in *Drosophila, C. elegans*, and mammalian cells [42,43,52]. We found that chronic arsenic exposure upregulated ALKBH4 protein and that ALKBH4 inhibition increased DNA 6mA levels in arsenic-transformed cells. Therefore, our data strongly suggest that in arsenic-transformed cells, the reduction in DNA 6mA methylation is mediated by ALKBH4 upregulation. We found that ALKBH4 inhibition decreased arsenic-induced tumorigenesis *in vitro* and *in vivo*. Our study also demonstrates that ALKBH4 has a pro-tumorigenic role in the arsenic-induced malignant transformation of keratinocytes. Previous data have shown that ALKBH4 functions to suppress colorectal cancer metastasis through competitive binding to WDR5 [70]. Conversely, ALKBH4 promotes tumorigenesis in non-small-cell lung cancer and correlates with poor prognosis [71]. These studies suggest that the function of ALKBH4 in cancer is dependent on context and may also be dependent on tissue type and stress.

Previous data showed strong 6mA enrichment in LINE-1 elements in the prefrontal cortex after stress and in embryonic stem cells [53]. Furthermore, 6mA has also been found to trigger the proteolysis of its cognate sensor proteins ASXL1 and MPND [52]. However, the role of 6mA on gene expression and in cellular homeostasis and stress responses remains enigmatic. ALKBH4 has been previously found to regulate histone methylation and serve as an epigenetic regulator [70,72]. Future investigation is warranted to determine how ALKBH4 regulates arsenic-induced skin tumorigenesis through DNA 6mA modification, whether changes in ALKBH4 or 6mA contribute to changes in histone methylations, and what the specific differences in 6mA methylation and targets are in response to chronic low arsenic exposure. While our previous study showed that m^6^A mRNA modification is decreased in arsenic-treated cells [55], our current data suggest that DNA 6mA, rather than m^6^A mRNA, may be the substrate of ALKBH4 in that ALKBH4 inhibition only affects DNA 6mA modification. Future studies are needed to determine other RNA species, including rRNA, tRNA, or other non-coding RNAs, may serve as potential ALKBH4 substrates.

Our lab previously found that arsenic can inhibit selective autophagy, which is a major process regulating protein stability [55]. RNA-seq data of arsenic-treated and control cells showed that the autophagy-related genes OPTN, LAPM1, p62, and ATG5 were decreased in arsenic-treated cells, compared with control cells [55]. Using BafnA1 to inhibit lysosomemediated autophagic degradation, we found that chronic arsenic exposure promoted ALKBH4 protein stability by impairing autophagic degradation, as evidenced by the unchanged ALKBH4 expression and LC-II accumulation in BafnA1-treated As-Tr cells. Future studies are required to further characterize the mechanism by which autophagy degrades ALKBH4 and identify the specific autophagy receptors responsible for ALKBH4 recruitment to the autophagosome.

## 5. Conclusions

In summary, we demonstrated that arsenic promotes ALKBH4 protein stability by impairing autophagy, which contributes to 6mA inhibition and malignant transformation and tumorigenesis. Our results further suggest that DNA 6mA modification serves as a new epigenetic mechanism in arsenic-induced skin carcinogenesis and may provide new insights into ALKBH4 and 6mA DNA methylation as potential novel therapeutic targets for the prevention and treatment of skin cancer in arsenic-exposed individuals.
